# An Ultrasensitive Electrochemical Biosensor for Nucleic Acid Detection Based on Silver Nanoflower-Stem-Loop Probes

**DOI:** 10.3390/s26165308

**Published:** 2026-08-21

**Authors:** Yingying Yuan, Xiaoyu Lei, Fengyu Li, Yuchen Su, Bo Liu, Lei Luo, Hangyu Zhang

**Affiliations:** 1 Department of Pediatric Gastroenterology, Women and Children’s Hospital of Dalian University of Technology, Dalian 116000, China; 2 Department of Oncology, Shengjing Hospital of China Medical University, Shenyang 110004, China; 3 Liaoning Key Laboratory of Integrated Circuit and Biomedical Electronic System, School of Biomedical Engineering, Faculty of Medicine, Dalian University of Technology, Dalian 116024, China

**Keywords:** electrochemical biosensor, stem-loop probe, silver nanoflowers, ultrasensitive detection, laser-induced graphene

## Abstract

**Highlights:**

**What are the main findings?**

- An AgNFs-SP-based biosensor achieves single-molecule-level nucleic acid detection.
- Dual-mode detection allows flexible trade-off between ultrahigh sensitivity (60 min, 1 aM) and rapid quantification (30 min, 0.1 fM).

**What are the implications of the main findings?**

- The signal amplification strategy is inherently versatile.
- The dual-mode design enables flexible adaptation to different application scenarios.

**Abstract:**

Nucleic acids are critical biomarkers that provide essential information throughout disease progression, making their detection critical to early diagnosis of both infectious and non-infectious diseases. However, existing detection methods, including classical analytical techniques and even most reported biosensors, are often constrained by complex procedures, high costs, and limited sensitivity, with the majority operating at the femtomolar level and failing to achieve single-molecule detection needed for early-stage diagnosis. Here, we report an electrochemical biosensor based on silver nanoflowers (AgNFs) integrated with stem-loop probes (SPs) for universal nucleic acid detection, using Norovirus RNA as a model target to validate the platform. The SPs serve as critical elements in a signal amplification system, converting target binding into a biotin–streptavidin recognition event, which leads to the accumulation of AgNFs-SP complexes on laser-induced graphene (LIG) electrodes and generates a strong electrochemical signal. Under optimized conditions with a 50 min hybridization incubation (total assay time ~60 min), the sensor exhibits a linear response to Norovirus RNA concentrations from 1 aM to 10 fM, with a measured detection limit of 1 aM, achieving single-molecule-level detection capability. For applications requiring faster turnaround, a 20 min hybridization incubation (~30 min total assay time) shifts the linear range to 0.1 fM–1 pM with a measured detection limit of 0.1 fM, offering more rapid quantification when the maximum sensitivity is not required. The proposed biosensor is cost-effective, amenable to miniaturization, and designed as a versatile platform adaptable to other nucleic acid targets by simply modifying the probe sequence, showing broad potential for early diagnosis of various diseases.

## 1. Introduction

Nucleic acids constitute the primary system for storing, replicating, and transmitting genetic information within living organisms [1,2]. They play indispensable roles in life processes and serve as crucial biomarkers for a spectrum of diseases, including both infectious diseases (e.g., COVID-19) and non-infectious diseases (e.g., cancer), by revealing extensive molecular-level genetic information [3,4]. Consequently, nucleic acid detection has become a cornerstone of early medical diagnosis [5], biomedical research [6] and pharmacological analysis [7]. For many diseases, particularly infectious ones, early diagnosis enables timely interventions that can slow disease progression, improve patient prognosis, and even save lives. However, detecting and capturing key nucleic acid biomarkers—such as viral RNA—remains a significant challenge due to their extremely low abundance in the early stages of infection [8].

To address this, a variety of strategies have been developed for nucleic acid detection. Classical analytical methods, including chromatography, mass spectrometry [2], DNA microarrays (gene chips) [9], and nucleic acid amplification techniques (e.g., PCR, RT-PCR) [10,11], have been widely used in laboratory settings. Despite their individual advantages, these methods are often constrained for point-of-care testing (POCT) applications due to complex and time-consuming procedures, high operational costs, and the need for specialized personnel [12,13]. Since the 19th century, biosensors—particularly electrochemical biosensors—have attracted significant research interest due to their ability to integrate multiple signal transduction and amplification mechanisms. Compared to fluorescent [14], colorimetric [15], and piezoelectric biosensors [16], electrochemical biosensors stand out for their high sensitivity, good stability, simple operation, potential for miniaturization, and cost-effectiveness [17,18,19,20].

Given the unique properties of nucleic acids—such as susceptibility to degradation, high sequence homology, and short base length—developing biosensors with effective signal amplification is imperative for trace nucleic acid detection [21,22,23,24,25]. Single-molecule quantification is essential for achieving such ultrasensitivity. To this end, various signal amplification strategies employing enzymes [26], electroactive substances [27], quantum dots [28], and nanomaterials [29] have gained significant attention and are increasingly utilized in electrochemical biosensors. In particular, nanoparticles have driven substantial progress in enhancing sensing performance. Their high surface-to-volume ratio and unique physicochemical properties enable them to function as highly efficient catalysts, significantly amplifying electrochemical signals from labeled molecules. Furthermore, their strong binding affinity for bioreceptors facilitates the development of highly sensitive biosensors [30,31]. For instance, Qian’s research group developed an ultrasensitive electrochemical platform for detecting human papillomavirus type 16 (HPV-16) DNA, achieving a linear detection range from 100 fM to 1 nM by using hollow covalent organic frameworks assembled with silver nanoparticle–DNA conjugates as signal labels, coupled with the CRISPR/Cas system [32]. In another study, a ratiometric electrochemical biosensor utilizing silver nanoparticles (AgNPs) incorporated with Walker amplification achieved a LOD as low as 3.2 × 10^−14^ M for microRNA [33]. While these silver nanoparticle-based systems have significantly improved sensitivity, most reported sensors still operate at the femtomolar (fM) level, leaving single-molecule detection a persistent challenge. To overcome this limitation, our research team has previously developed a nanostructured silver material, termed silver nanoflowers (AgNFs). These particles feature a flower-like spherical morphology with abundant surface protrusions, demonstrating a capacity to generate strong and stable electrochemical signals [34].

Leveraging this property, here we integrate AgNFs with stem-loop probes (SPs) to construct a simple, ultrasensitive electrochemical biosensor for universal nucleic acid detection. To validate the platform, we employ Norovirus RNA as a clinically relevant model target. Unlike previously reported amplification-based electrochemical biosensors, the proposed AgNFs-SPs platform enables two modes of quantitative detection: single-molecule-level detection is achieved with a 50 min hybridization incubation, and more rapid quantification within 20 min when the maximum sensitivity is not required. The sensing platform is constructed by conjugating SPs to AgNFs and immobilizing capture probes onto laser-induced graphene (LIG) electrodes. The 3’ end of the SPs is labeled with biotin. Upon specific hybridization with the target nucleic acid (Norovirus RNA in this study), the SPs undergo a conformational change that opens the stem-loop structure, thereby exposing the biotin tag. This exposed biotin then binds via strong non-covalent interaction to streptavidin pre-immobilized on the electrode surface. This recognition event leads to the accumulation of numerous AgNFs-SPs complexes near the electrode, generating a significantly amplified electrochemical signal. Under optimized conditions using Norovirus RNA as the model target, the sensor exhibits a wide linear detection range from 1 aM to 10 fM with the 50 min protocol, achieving a measured detection limit of 1 aM and demonstrating its ultrasensitive detection capability. The platform’s versatility is inherent in its design: the stem-loop probe sequence can be readily reprogrammed to recognize different nucleic acid targets, making this approach adaptable to a wide range of diagnostic applications.

## 2. Materials and Methods

### 2.1. Reagents and Apparatus

All HPLC-purified oligonucleotide sequences were synthesized by Sangon Biotech Co., Ltd. (Shanghai, China): Norovirus RNA (5′-AUGAAGAUGGCGUCGAGUG-3′), 3 mismatch Norovirus RNA sequence (5′-AUGAAGAUUGCUUCGAUUG-3′), and the SP specific to Norovirus RNA (5′-NH_2_C_6_-GGGAACACTCGACGCCATCTTCATTTCCC-biotin-3′). TE buffer (1× Solution, low EDTA, pH 8.0) and DEPC-treated water were also obtained from Sangon Biotech Co., Ltd. (Shanghai, China). Bovine serum albumin (BSA) was purchased from Shanghai Aladdin Biochemical Technology Co., Ltd. (Shanghai, China). N,N-Dimethylformamide (DMF) was purchased from Shanghai Macklin Biochemical Technology Co., Ltd. (Shanghai, China). N-Hydroxysuccinimidyl pyrenebutanoate (PBASE) was obtained from Shanghai Bide Pharmatech Co., Ltd. (Shanghai, China). Glutaraldehyde (GA) was purchased from Shanghai Yien Chemical Technology Co., Ltd. (Shanghai, China). 4-Aminobenzenethiol (4-ATP) was purchased from Shanghai Haohong Biomedical Technology Co., Ltd. (Shanghai, China). All aqueous solutions were prepared with deionized water (18.25 MΩ·cm) obtained from an ultra-pure water generator (Canshi, Canmei Electric Co., Ltd., Cixi, Zhejiang Province, China).

All electrochemical measurements, including differential pulse voltammetry (DPV), electrochemical impedance spectroscopy (EIS), and cyclic voltammetry (CV), were performed using a CHI660e electrochemical analyzer (Shanghai Chenhua Instrument Co., Ltd., Shanghai, China). The surface morphology of the samples was examined using a scanning electron microscope (SEM, MIRA3-LMH, TESCAN, Brno, Czech Republic) at the acceleration voltage of 20 kV. The crystal structure of the samples was characterized using an X-ray diffractometer (XRD, D8 Advance, Bruker, Karlsruhe, Germany) over a wide angle range of 5–120°. Concurrently, X-ray photoelectron spectroscopy (XPS, ESCALAB XI, Thermo Fisher Scientific, Waltham, MA, USA) was employed to analyze the surface elemental composition and chemical state of AgNFs in order to verify their successful preparation.

### 2.2. Preparation of AgNFs

AgNFs were synthesized following the method previously reported by our research group [34]. Briefly, 1 mL of 0.5 M AgNO_3_ solution and 0.1 mL of 0.25 M citric acid solution were rapidly added to 10 mL of ultrapure water maintained in an ice bath at 4 °C. The mixture was vigorously stirred at 15,000 rpm for 15 min using a magnetic stirrer. Subsequently, 0.5 mL of 1 M ascorbic acid solution was introduced, and stirring continued for an additional 15 min, during which the solution gradually turned gray. The resulting suspension was centrifuged at 10,000 rpm for 2 min, and the pellet was washed several times with ultrapure water. After drying at 60 °C, the obtained AgNFs were stored at 4 °C in the dark until use.

### 2.3. Synthesis of AgNFs-SPs

First, 10 mg of AgNFs were dispersed in a 4-ATP solution prepared by dissolving 50 μL of 4-ATP in 1 mL of ethanol. The mixture was stirred vigorously for 12 h to obtain a uniformly black suspension, then centrifuged at 10,000 rpm for 10 min. The precipitate was washed repeatedly with 75% ethanol and ultrapure water, redispersed in 1 mL of ultrapure water, and sonicated for 1 min using an ultrasonic probe (tip diameter 2 mm) operated at 1% of maximum power (900 W) with a pulse cycle of 3 s on and 9 s off. During this process, the thiol group of 4-ATP formed a coordination bond (Ag–S) with the AgNFs, yielding the 4-ATP/AgNFs complex. Glutaraldehyde (GA), a common crosslinker, was then introduced. Specifically, 1 mL of 50% GA solution was added to the 4-ATP/AgNFs suspension and stirred vigorously for 12 h. One aldehyde group of GA covalently coupled with the free amino group on 4-ATP/AgNFs, while the other aldehyde moiety remained available for subsequent functionalization. The mixture was centrifuged twice at 10,000 rpm for 10 min and washed with ultrapure water, and the pellet was redispersed in 2 mL of ultrapure water by brief sonication (1 min) to obtain GA/4-ATP/AgNFs. To construct the capture probe, 60 μL of SP and 1 mL of 10 mg/mL BSA were simultaneously added to the GA/4-ATP/AgNFs suspension and stirred magnetically for 4 h. The SP is designed with a loop region complementary to the target Norovirus RNA and a stem region containing biotin at the 3′ end for specific binding to streptavidin on the electrode surface. The free aldehyde groups on the AgNF surface reacted with the amino group at the 5′ end of the SP, ultimately forming the AgNFs-SP conjugates. The prepared conjugates were stored at 4 °C until use.

### 2.4. Fabrication of LIG Electrode

LIG electrodes were fabricated using a laser engraving machine (power: 14%, depth: 14%) to pattern a precursor material consisting of polyimide (PI) tape (100 μm thickness) attached to a phenolic resin plate. Prior to engraving, the PI tape was cleaned with 75% ethanol and ultrapure water to remove surface impurities and ensure a clean, flat substrate. The electrode pattern measured 12 mm × 17 mm, with a working electrode diameter of 3 mm. Conductive copper tape was attached to the printed LIG electrodes to facilitate electrochemical connections. The reference electrode area was then coated with Ag/AgCl paste to establish a stable reference potential. After drying the Ag/AgCl layer, a stack consisting of a release film, thermoplastic polyurethane (TPU), and the LIG electrode was assembled and hot-pressed to encapsulate the device. During encapsulation, the working electrode area remained exposed, as illustrated schematically in Figure S1.

### 2.5. Surface Modification of the Electrochemical Biosensor

The bare LIG electrode was rinsed 2–3 times with 75% ethanol and ultrapure water prior to modification. Then, 4 μL of 40 mM PBASE dissolved in DMF was drop-cast onto the working electrode surface. The pyrene moiety of PBASE enables non-covalent π–π stacking with graphene, facilitating biomolecule immobilization. After 1 h of incubation, the electrode was rinsed sequentially with DMF and ultrapure water. Next, 6 μL of 0.1 mg/mL streptavidin solution was applied and allowed to react for 1 h. The amino groups of streptavidin formed covalent amide bonds with the activated ester groups of PBASE, enabling subsequent capture of biotin-labeled AgNFs-SP conjugates. The electrode was thoroughly rinsed with ultrapure water, followed by incubation with 10 μL of 10 mg/mL BSA solution for 1 h to block nonspecific adsorption sites. Finally, the electrode was rinsed again with ultrapure water. All incubation steps were performed in a humidity-controlled chamber maintained at 90% relative humidity and 25 °C.

### 2.6. Electrochemical Measurements

The target Norovirus RNA was diluted in TE buffer (1×, low EDTA, pH 8.0). Prior to electrochemical detection, the AgNFs-SP solution and Norovirus RNA target solution were mixed at a volume ratio of 1:1.25 in a centrifuge tube and incubated for 1 h at room temperature to form the target/AgNFs-SP complex. During this step, the target RNA hybridized with the complementary loop region of the stem-loop probe, forming a stable double-stranded structure. Subsequently, 5 μL of the target/AgNFs-SP complex was drop-cast onto the surface of the modified LIG electrode. After 10 min of incubation, the electrode was rinsed with ultrapure water to remove non-specifically bound material.

The LIG electrode was then connected to the CHI660e electrochemical workstation. All electrochemical measurements were performed using a conventional three-electrode system. DPV measurements were carried out in 100 μL of 0.1 M KCl solution under the following parameters: potential window from −0.2 V to +0.2 V, pulse amplitude 0.05 V, step increment 0.004 V, pulse width 0.05 s, pulse period 0.5 s, and quiet time 2 s. EIS was conducted in a solution containing 5 mM [Fe(CN)_6_]^3−^/^4−^ and 1 M KCl, with a frequency range from 100 kHz to 0.1 Hz. CV measurements were performed in the same [Fe(CN)_6_]^3−^/^4−^ solution (5 mM in 1 M KCl) with a potential sweep from −0.3 V to +0.3 V at a scan rate of 0.1 V/s.

## 3. Results and Discussion

### 3.1. The Construction and the Principle of the Biosensor

**Figure 1:**
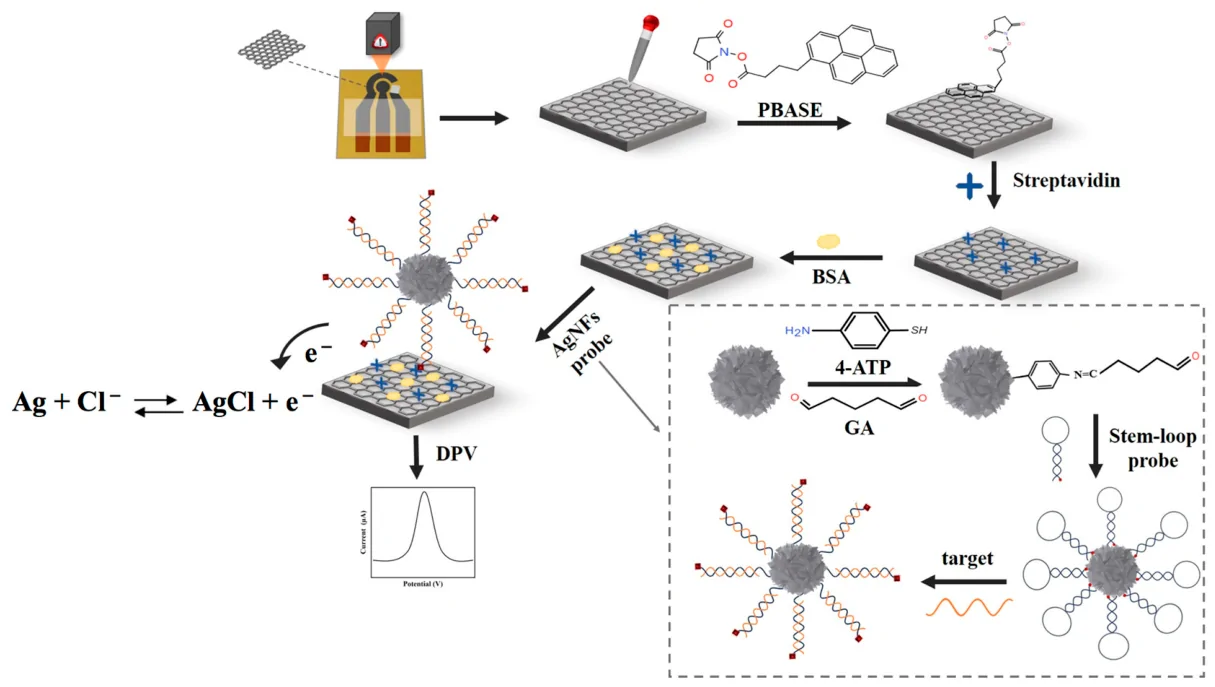
Schematic of electrochemical biosensor preparation and target detection.

The fabrication process and the detection principle of the biosensor are illustrated in Figure 1. Streptavidin is first immobilized on LIG electrode via π–π stacking between the graphene surface and the pyrene moiety of PBASE. On the other hand, SPs are conjugated to AgNFs through Ag–S bonds to form AgNFs-SP complexes in solution. In the presence of the target Norovirus RNA, hybridization with the loop region of the SP induces a conformational change that opens the stem-loop structure, exposing the biotin label at the 3′ end. The high-affinity biotin–streptavidin interaction then enables the capture of abundant AgNFs-SP complexes onto the electrode surface, generating a significantly amplified electrochemical signal proportional to the target concentration. In the absence of target, no substantial signal is observed. Ultrasensitive detection of Norovirus RNA is thus achieved by measuring the current response before and after target introduction.

### 3.2. Characteristics of AgNFs-SPs

**Figure 2:**
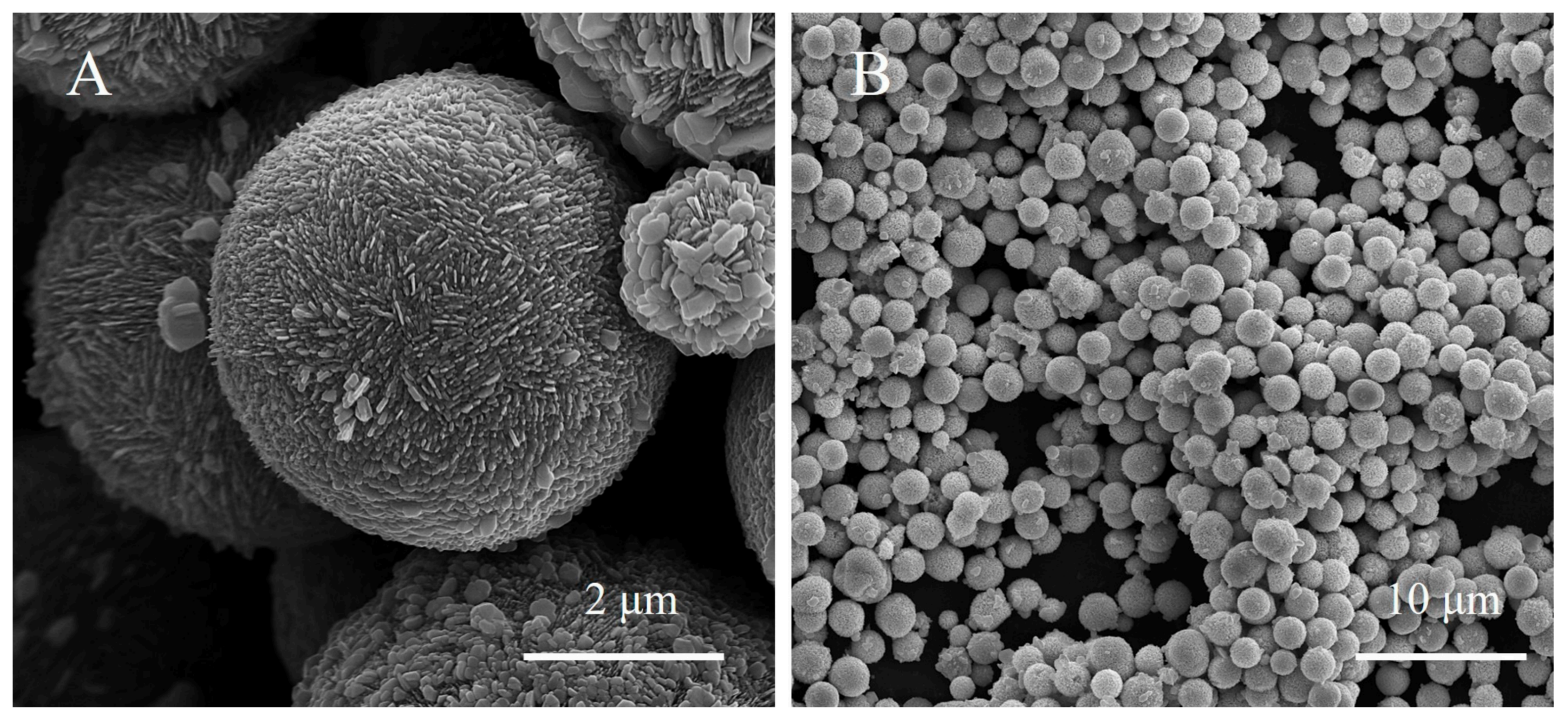
SEM images of AgNFs. (**A**) A high-magnification image showing detailed surface morphology. (**B**) A low-magnification image revealing the overall size distribution and uniformity.

AgNFs with an average diameter of 3.5 μm were synthesized via chemical reduction using citric acid and ascorbic acid. SEM revealed a near-spherical morphology with densely assembled petal-like silver layers (Figure 2).

**Figure 3:**
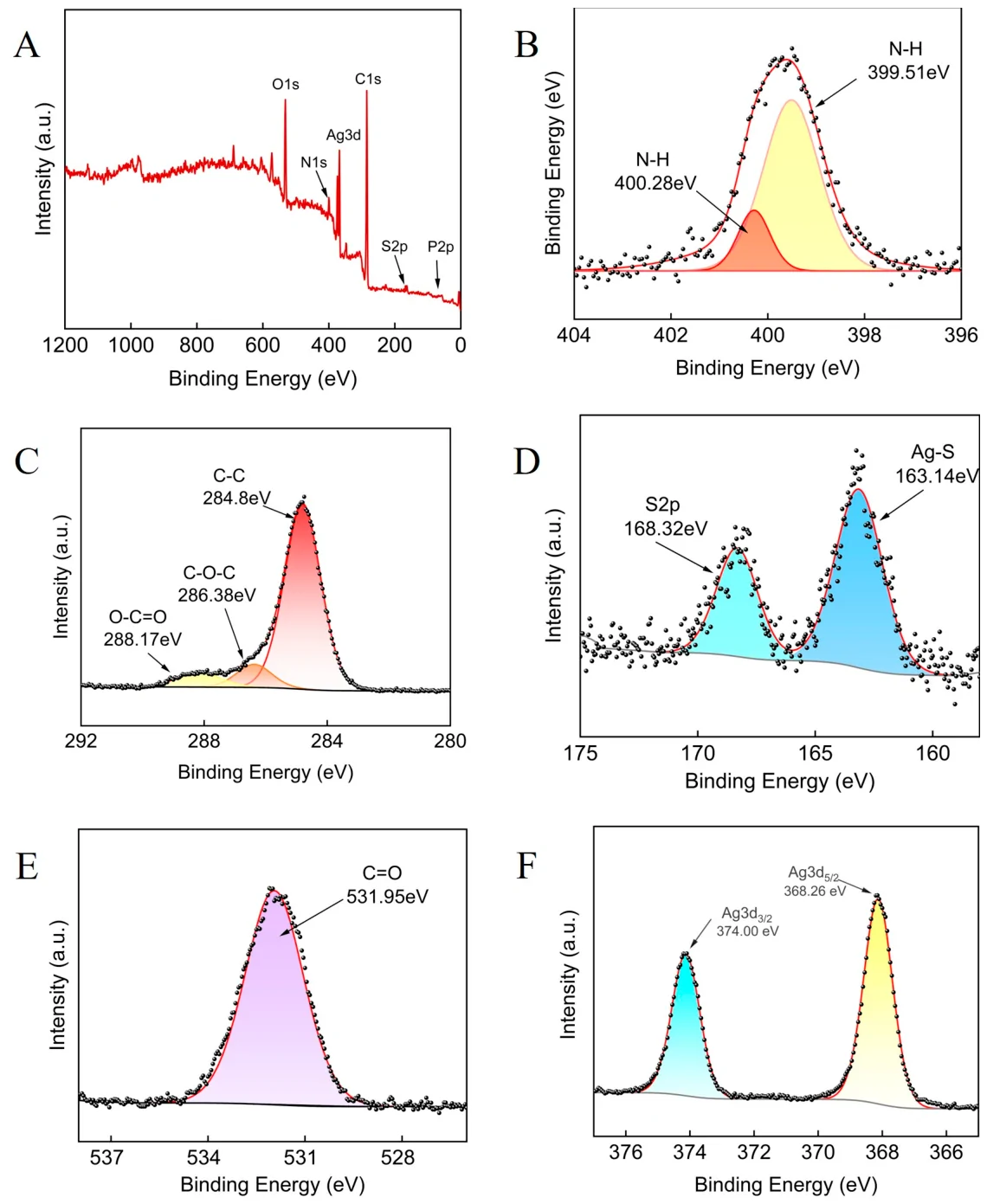
XPS spectra of AgNFs-SP: survey spectrum (**A**), high-resolution N 1s spectrum (**B**), C 1s spectrum (**C**), S 2p spectrum (**D**), O 1s spectrum (**E**), and Ag 3d spectrum (**F**).

XPS was employed to analyze the surface composition of the AgNFs-SPs (Figure 3). The full survey spectrum exhibited distinct peaks corresponding to Ag 3d, C 1s, N 1s, O 1s, S 2p, and P 2p, confirming the presence of these elements. During conjugate preparation, 4-ATP was first introduced to functionalize the AgNFs via its thiol group, while its amino group remained available for further coupling. GA was then added as a crosslinker, with one aldehyde group reacting with the amino group of 4-ATP. Finally, BSA and SPs were conjugated to the AgNF surface. The higher relative concentrations of C, N, and O compared to S and P are consistent with the elemental compositions of BSA (containing C, H, N, O, S) and SPs (containing H, C, N, O, P). These spectral features collectively confirm the successful stepwise conjugation of 4-ATP, GA, BSA, and SPs to the AgNFs.

XRD was performed to characterize the crystalline structure of the AgNFs (Figure S2). Diffraction peaks observed at approximately 38.1°, 44.3°, 64.4°, 77.4°, and 81.6° correspond to the (111), (200), (220), (331), and (222) crystal planes of face-centered cubic (fcc) silver, respectively, in good agreement with standard reference patterns [35].

### 3.3. Characterization of the Biosensor Interface

**Figure 4:**
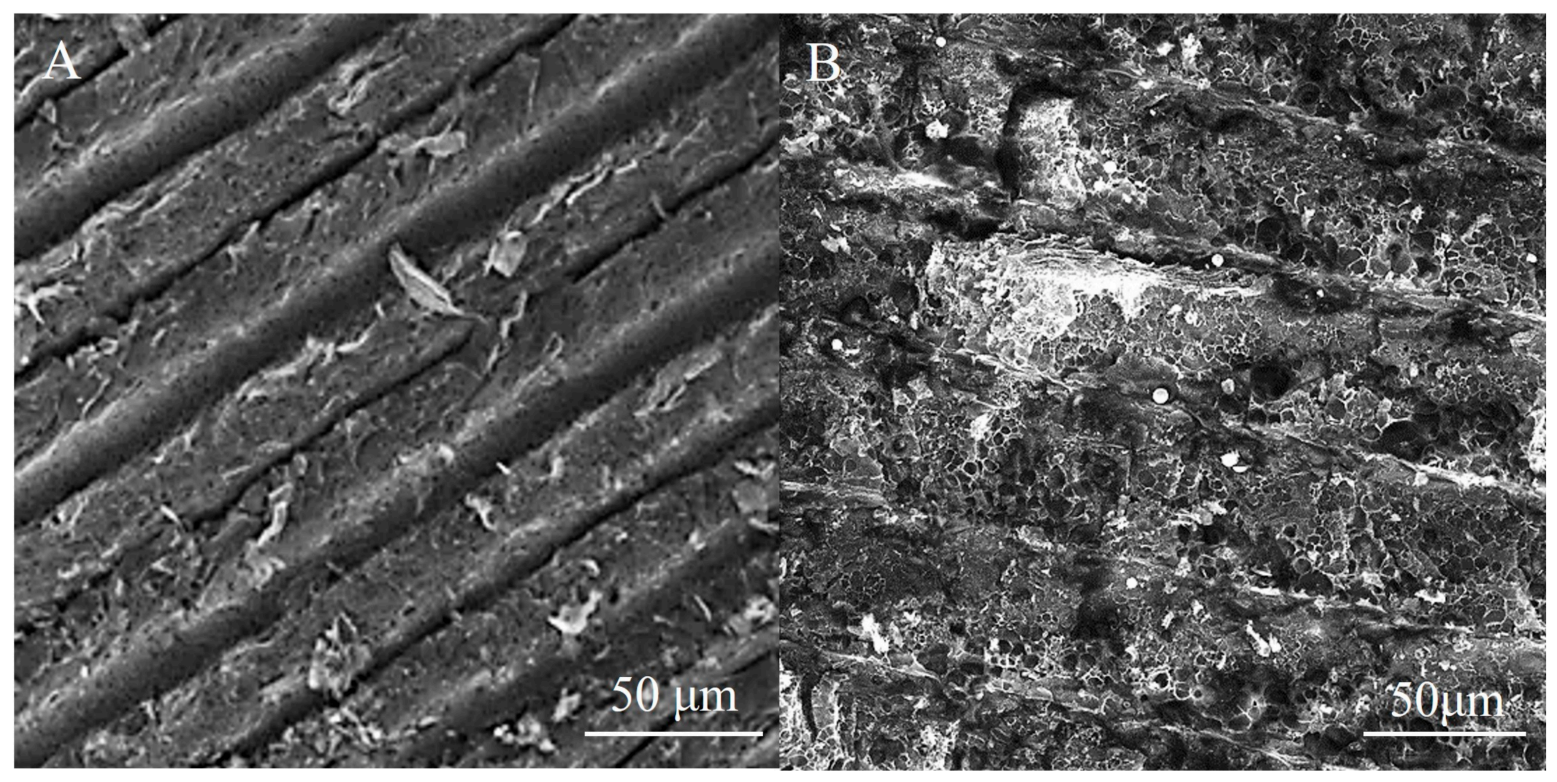
SEM images of LIG electrodes. (**A**) A typical SEM image of bare LIG electrode. (**B**) A typical SEM image of LIG electrodes after capturing AgNFs-SPs.

The surface morphology of the LIG electrode before and after target detection was examined by SEM. The bare LIG electrode exhibits a three-dimensional porous network structure (Figure 4A), resulting from laser-induced depolymerization, carbonization, and graphitization of the polyimide precursor. During this process, the interaction of atmospheric oxygen and water vapor with the forming graphite promotes the generation of carbon monoxide (CO) and carbon dioxide (CO_2_); the release of these gases contributes to the development of the porous architecture [36]. After detection, abundant AgNFs-SP complexes captured on the electrode surface via the streptavidin–biotin system are clearly observed (Figure 4B), confirming successful target-induced accumulation of signal tags.

**Figure 5:**
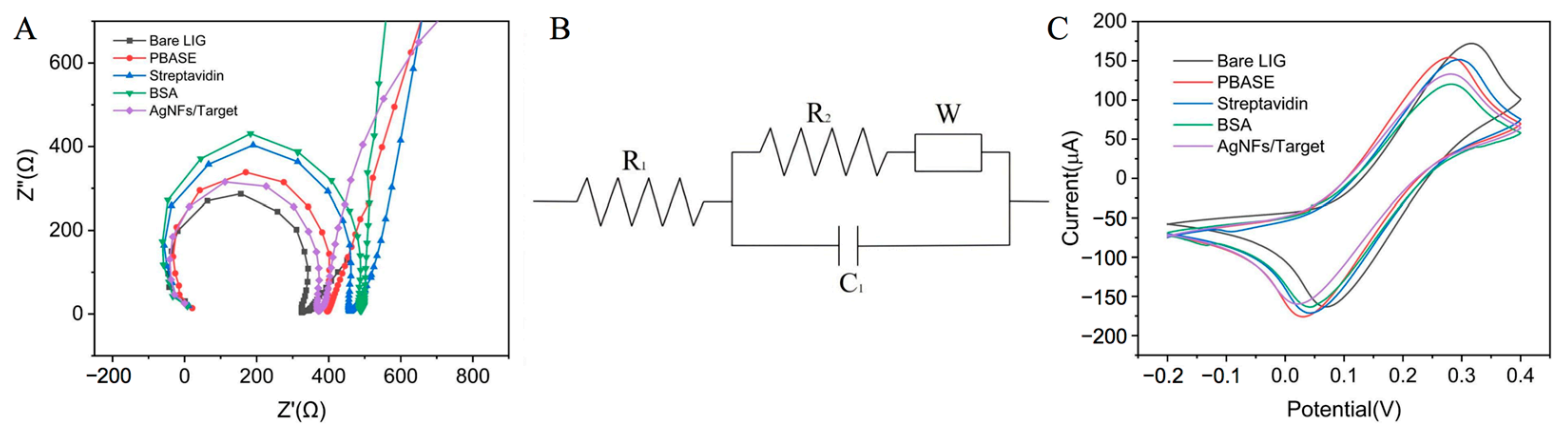
Electrochemical characterization of the biosensor fabrication and detection process. (**A**) Nyquist plot of EIS recorded for the stepwise modification of the working electrode and the detection process. (**B**) Equivalent circuit diagram for EIS. (**C**) CV curves obtained under the same condition.

EIS and CV were employed to characterize both the stepwise fabrication of the biosensor and its response upon target capture. Measurements were performed in a solution containing 5.0 mM [Fe(CN)_6_]^3−^/^4−^ and 0.1 M KCl, using [Fe(CN)_6_]^3−^/^4−^ as a redox probe. Figure 5A presents the Nyquist plots recorded during sequential modification and subsequent detection. In these plots, the semicircle diameter corresponds to the charge transfer resistance (Rct). The bare LIG electrode exhibited a small semicircle (black curve). After drop-casting PBASE, the semicircle radius increased slightly. Subsequent modification with streptavidin led to a more pronounced increase in Rct, attributed to hindered access of the redox probe by the biomolecular layer. Blocking with BSA, a non-conductive protein, further increased Rct. Notably, after capture of AgNFs-SP conjugates via target-induced biotin–streptavidin interaction, Rct decreased significantly. This decrease indicates that the highly conductive AgNFs facilitate electron transfer between the redox probe and the electrode, thereby enhancing biosensor sensitivity. CV measurements provided complementary evidence (Figure 5B). The bare LIG electrode displayed a well-defined, symmetrical redox pair of [Fe(CN)_6_]^3−^/^4−^. Progressive modification resulted in gradually decreasing redox peak currents, consistent with increased electron transfer distance and hindered probe diffusion. Following target-induced capture of AgNFs-SP conjugates, the current response increased substantially, confirming enhanced electrode conductivity conferred by the AgNFs. These results collectively demonstrate successful stepwise construction of the biosensing interface and confirm that target recognition leads to effective capture of conductive signal tags, producing a measurable electrochemical response. To further support this interpretation, the R_ct_ values obtained from fitting the EIS data (using the Randles-type equivalent circuit shown in Figure 5B), along with the CPE parameters and the CV peak currents (I_ox_ and I_red_), are summarized in Table S1. The R_ct_ values increase progressively from the bare LIG electrode (317 Ω) to the BSA-blocked electrode (470 Ω), confirming the successful stepwise formation of the insulating biomolecular layer. After target-induced capture of AgNFs-SP conjugates, R_ct_ decreases significantly to 342 Ω, consistent with the conductive nature of AgNFs facilitating electron transfer. The CV peak currents follow a similar trend: I_ox_ decreases from 135.8 μA to 92.3 μA upon modification and recovers to 99.2 μA after target capture. Although the magnitudes differ between the two techniques, reflecting their different sensitivities to interfacial changes, the overall trends are fully consistent and collectively confirm the successful construction of the biosensing interface.

### 3.4. Optimization of Experimental Conditions

**Figure 6:**
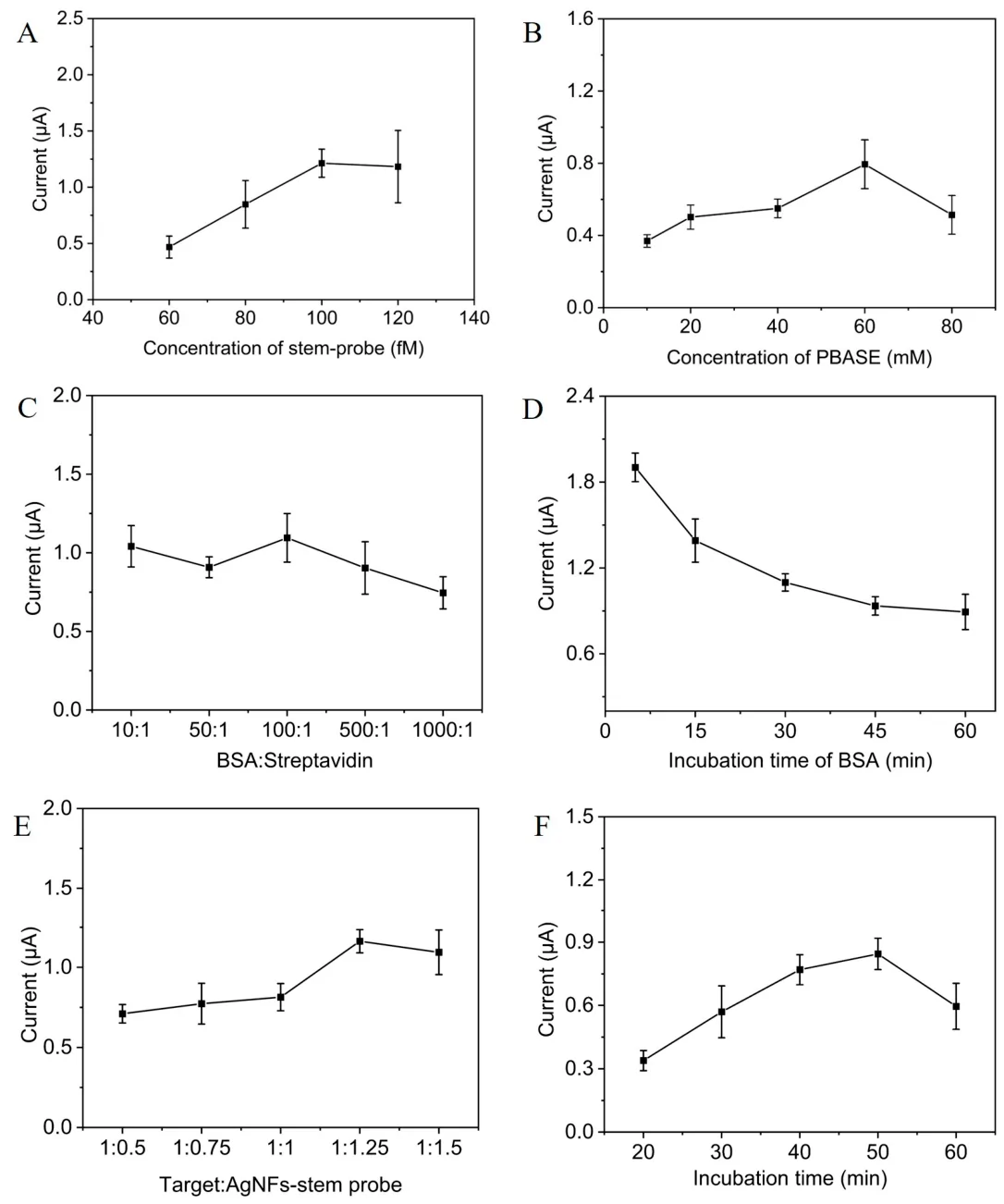
Optimization of preparation parameters of the electrochemical biosensor (*n* = 4). (**A**) Optimization of stem-loop probe dosage; (**B**) optimization of PBASE concentration; (**C**) optimization of the BSA-to-streptavidin concentration ratio; (**D**) optimization of BSA modification time; (**E**) optimization of the target-to-probe incubation ratio; (**F**) optimization of target-to-probe incubation time.

To achieve optimal analytical performance, key experimental parameters including SP dosage, PBASE concentration, BSA-to-streptavidin ratio, BSA blocking time, target-to-probe ratio, and hybridization time were systematically investigated (Figure 6). The amount of SP conjugated to the AgNF surface directly influences target recognition efficiency. As shown in Figure 6A, the electrochemical signal increased with SP dosage from 60 to 100 pmol, reaching a maximum at 100 pmol. Therefore, 100 pmol was selected as the optimal SP dosage. PBASE concentration affects the density of streptavidin immobilization on the electrode surface.

PBASE concentrations of 10, 20, 40, 60, and 80 mM were evaluated (Figure 6B). The current response increased progressively up to 60 mM, beyond which further increase led to signal decline. Thus, 60 mM PBASE was chosen for subsequent experiments.

To optimize the electrode surface composition, BSA was mixed with streptavidin at various ratios while maintaining a fixed streptavidin concentration of 0.1 mg Ml^−1^ (Figure 6C). Given the relatively large size of the AgNFs (~3.5 μm), complete coverage of the electrode surface with streptavidin is unnecessary; instead, a mixed layer comprising a small amount of streptavidin and a larger proportion of BSA—a commonly used and cost-effective blocking protein—was employed to capture biotin-labeled AgNFs-SP conjugates while minimizing nonspecific adsorption. The electrochemical response was evaluated at BSA-to-streptavidin ratios of 10:1, 50:1, 100:1, 500:1, and 1000:1. Comparable signal intensities were observed at ratios up to 100:1, with a slight decrease at 500:1 and further reduction at 1000:1. Given that an additional BSA physical adsorption step was subsequently applied for antifouling, the observed differences among lower ratios likely reflect normal experimental variability. The modest signal decline at the highest BSA ratios (500:1 and 1000:1) may indicate that the density of covalently immobilized streptavidin has been reduced below a threshold required for efficient capture of the biotinylated AgNFs-SP conjugates, even though ample biotin-binding capacity might still be expected. Alternatively, at extremely high BSA ratios, the local environment surrounding the remaining streptavidin molecules may be sterically crowded by BSA, potentially hindering access of the bulky AgNFs-SP complexes, although further investigation would be required to confirm this mechanism. Based on these results, and considering both signal intensity and reagent economy, the BSA-to-streptavidin ratio of 100:1 was selected for subsequent experiments.

BSA blocking time was optimized to minimize nonspecific adsorption. Incubation times of 5, 15, 30, 45, and 60 min were tested (Figure 6D). Insufficient blocking at 5 min resulted in elevated background interference. As blocking time increased, the current gradually declined and stabilized after 45 min, indicating effective surface passivation. Accordingly, 45 min was selected as the optimal blocking duration.

The incubation ratio between target Norovirus RNA and AgNFs-SP conjugates was optimized by varying the amount of AgNFs-SP added while maintaining a fixed target concentration at 10 fM (Figure 6E). The current response increased with increasing AgNFs-SP input until meeting a target-to-probe ratio of 1:1.25, beyond which signal saturation or steric hindrance effects likely occurred. This ratio was therefore selected for subsequent experiments.

Hybridization time between AgNFs-SP and target RNA critically affects signal intensity. As shown in Figure 6F, the electrochemical signal increased progressively from 20 to 50 min, reaching a maximum at 50 min, followed by a noticeable decrease at 60 min. The initial signal increase reflects progressive hybridization between the target RNA and the loop region of the stem-loop probes, leading to increasing capture of AgNFs-SP complexes on the electrode surface. Extended hybridization time may promote nonspecific interactions or partial displacement of weakly bound complexes. At longer incubation times, the high local concentration of hybridized complexes could induce steric repulsion between adjacent AgNFs-SPs, potentially destabilizing some of the captured complexes or altering their conformation in a manner that reduces effective electron transfer. Based on the time-dependent profile showing optimal signal at 50 min followed by decline at 60 min, 50 min was selected as the optimal hybridization duration for subsequent experiments, balancing maximum signal intensity with assay throughput.

Overall, the observed trends in the optimization experiments provide empirical guidance for selecting optimal parameters, and the mechanistic interpretations offered above are supported by previously reported phenomena in similar nucleic acid-based electrochemical sensing systems.

### 3.5. Analytical Performance

**Figure 7:**
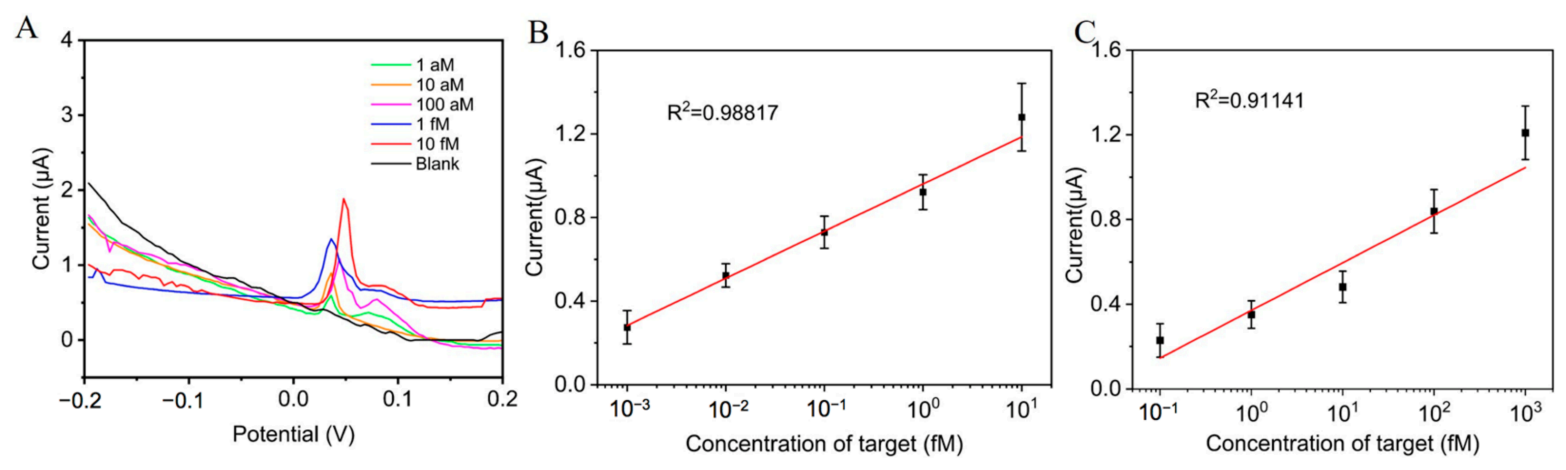
Electrochemical detection performance of the biosensor for Norovirus RNA. (**A**) DPV curves obtained at various concentrations of Norovirus RNA after 50 min hybridization incubation. (**B**) Corresponding calibration curve showing current response as a function of Norovirus RNA concentration (on a logarithmic scale) after 50 min incubation (Δ*I* = 0.961 lg*C_RNA_* + 0.226). (**C**) Calibration curve after 20 min hybridization incubation, with current response versus logarithmic concentration of Norovirus RNA (Δ*I* = 0.371 lg*C_RNA_* + 0.225).

Under optimized conditions, the analytical performance of the electrochemical biosensor for Norovirus RNA detection was evaluated (Figure 7, *n* = 5). As shown in Figure 7A, the peak current of the sensor increased with the concentration of the Norovirus RNA being measured. A small additional peak at ~0.075 V was occasionally observed in some DPV curves, attributed to silver oxidation in chloride-containing electrolyte. This minor feature did not affect the quantitative analysis, which was based on the main peak at ~0.04 V. After 50 min hybridization incubation followed by 10 min electrode incubation, the DPV current response exhibited a good linear relationship with the logarithm of Norovirus RNA concentration from 1 aM to 10 fM (Figure 7B). The linear regression equation was Δ*I* = 0.961 lg*C_RNA_* + 0.226 (R^2^ = 0.98817), with a measured detection limit of 1 aM. At this concentration, approximately three Norovirus RNA molecules are present in the 5 μL test solution, indicating that the biosensor, enabled by the efficient signal amplification of AgNFs, is capable of single-molecule-level detection. We use the term ‘single-molecule-level detection’ to indicate that the detection limit corresponds to a concentration at which the number of target molecules in the test sample is on the order of a single molecule (approximately three molecules at 1 aM in 5 μL). We acknowledge that this does not constitute true single-molecule detection at the individual event level, which would require different experimental configurations. Both the linear range and the measured detection limit compare favorably with previously reported nucleic acid detection assays (Table 1). To meet the demand for more rapid testing, a shortened hybridization incubation time of 20 min was also investigated (Figure 7C). Under this condition, the logarithmic concentration exhibited a linear relationship with current response from 0.1 fM to 1 pM, with regression equation Δ*I* = 0.371 lg*C_RNA_* + 0.225 (R^2^ = 0.91141) and a measured detection limit of 0.1 fM. Notably, this represents a shift in the linear range by approximately two orders of magnitude toward higher concentrations compared to the 50 min assay, reflecting the expected trade-off between assay speed and sensitivity. The 20 min hybridization yields an overall detection time of approximately 30 min (including the subsequent 10 min electrode incubation), offering a faster alternative when maximum sensitivity is not required. Both incubation protocols are quantitative, with the choice between them depending on the specific application requirements: the 50 min protocol provides ultrasensitive detection down to the single-molecule level, while the 20 min protocol enables more rapid quantification with acceptable sensitivity for samples with higher target concentrations. To assess batch-to-batch reproducibility, three independently prepared batches of sensors were tested under identical conditions (Figure S3). The inter-batch relative standard deviation (RSD) was 10.38% (*n* = 5), indicating that the fabrication protocol yields reasonably reproducible devices.

**Figure 8:**
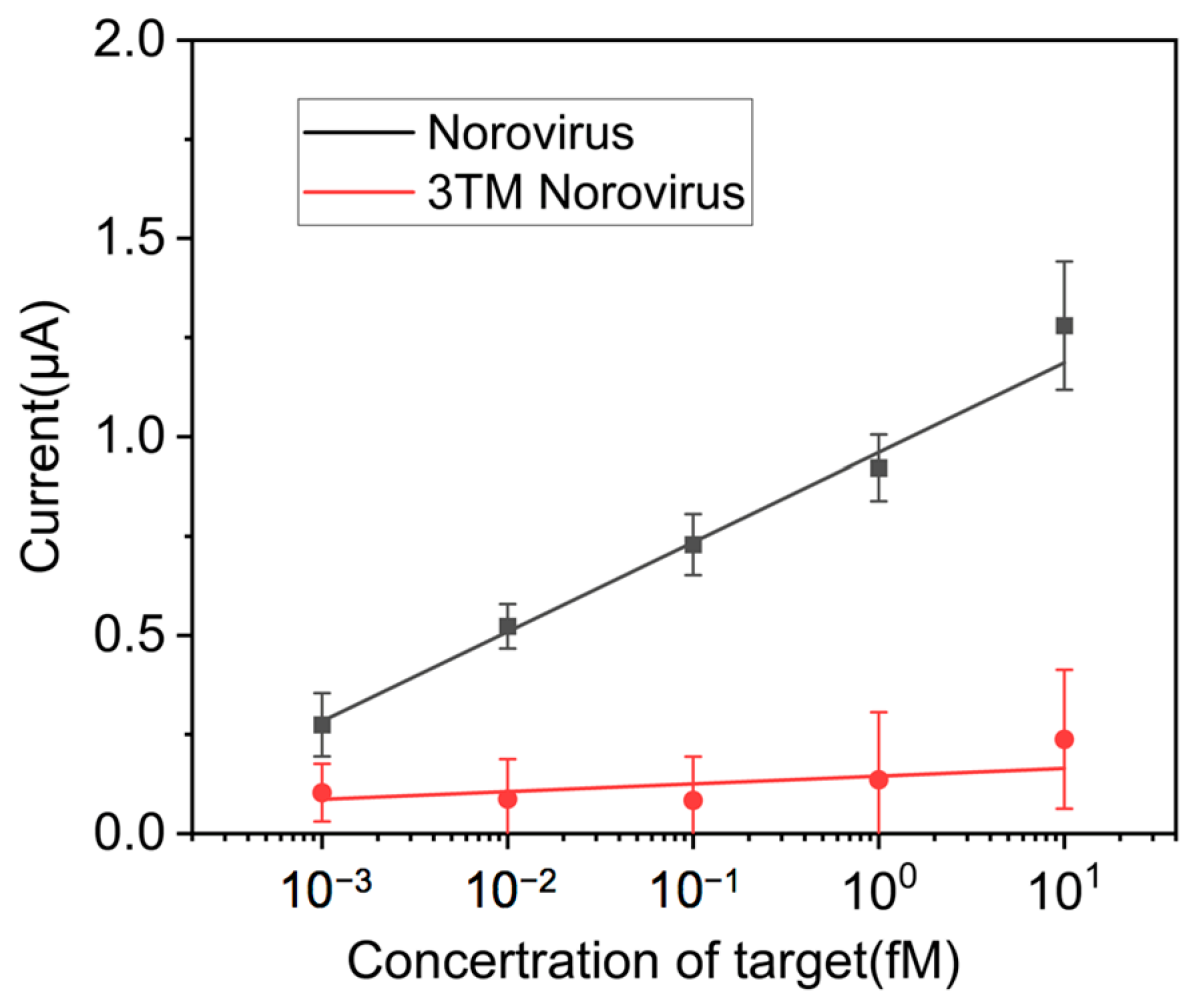
Comparison of calibration curves for the fully complementary Norovirus RNA target and a three-base mismatch sequence. The mismatch sequence consistently exhibited an approximately five-fold lower current response than the complementary target across the tested concentration range, confirming the high sequence specificity of the electrochemical biosensor. Error bars represent the standard deviation of four replicate measurements (*n* = 4).

The specificity of the sensor was rigorously evaluated using both a blank control and a three-base mismatch sequence. As shown in Figure 7A, the blank control (in the absence of target) produced no significant current response, confirming negligible non-specific adsorption. Furthermore, under identical conditions, the DPV current response for the fully complementary Norovirus RNA target was approximately five-fold higher than that for the three-base mismatch sequence across the entire concentration range tested (Figure 8). This significant signal difference demonstrates the excellent single-base discrimination capability of the stem-loop probe-based biosensor.

## 4. Conclusions

In conclusion, we have developed an ultrasensitive electrochemical biosensor based on silver nanoflower-stem-loop probe conjugates (AgNFs-SP) for nucleic acid detection. The biosensor leverages the large surface area and excellent conductivity of AgNFs for signal amplification, combined with the conformational switching of stem-loop probes for specific target recognition. Using Norovirus RNA as a model target to validate the platform, the sensor achieves a linear detection range from 1 aM to 10 fM with a measured detection limit of 1 aM under optimized conditions with a 50 min hybridization incubation (total assay time ~60 min), corresponding to single-molecule-level detection capability. For applications prioritizing speed, a 20 min hybridization incubation (~30 min total assay time) provides a linear range of 0.1 fM to 1 pM with a measured detection limit of 0.1 fM. The key features of this biosensor platform include: (i) the integration of AgNFs with stem-loop probes for nucleic acid detection, which enables attomolar-level sensitivity through high-density signal labeling; (ii) the stem-loop probe design, which allows direct target-induced biotin exposure without enzymatic amplification; and (iii) dual-mode quantification for either ultrahigh sensitivity or faster detection. The stem-loop probe sequence can be readily reprogrammed to recognize different nucleic acid targets, making the approach adaptable to a wide range of diagnostic applications. While the primary objective of this work was to establish the analytical performance of the AgNFs-SP signal amplification strategy in a controlled environment, we recognize that practical diagnostic applications will require further validation in complex biological fluids. Nonetheless, with its cost-effectiveness, miniaturization potential, and excellent analytical performance, the proposed biosensor represents a promising platform for early diagnosis and monitoring of various diseases.

## Figures and Tables

**Table 1:** Performance comparison of electrochemical sensors for the detection of nucleic acid biomarkers.

Target	Signal Amplification Material	Electrodes	Detection Methods	Linear Range	Detection Limit	Ref
Norovirus RNA	AgNFs-SP	LIG	DPV	1 aM–10 fM	1 aM	This work
DENV ^a^	TNM-HCR ^b^	Gold electrode	CA ^c^	1.6–1000 pM	188 fM	[37]
HPV-16 DNA	CRISPR/Cas12a, HCOFs ^d^	Gold electrode	EIS	100 fM–1 nM	57.2 fM	[32]
HBV DNA ^e^	CoCu/CNC/Pt ^f^ nanozymes	Gold electrode	CA	100 aM–10 nM	0.09 fM	[38]
MiRNA-21	Mxene ^g^-AuPd	MXene-rGO-Au modified GCE ^h^	DPV	1 fM–1 nM	0.42 fM	[39]
MiRNA-205-5p	CHA-HCR	Au NPs/SPCE ^i^	LSV ^j^	0.1 pM–10 μM	28 fM	[40]
MiRNA-21	PdNPs ^k^	Gold electrode	DPV	10 fM–1 nM	3.33 fM	[41]
MiRNA-141	functionalized PNA ^l^	LIG	DPV	0.01–100 fM	0.6 aM	[42]
MiRNA-21	Q-walker ^m^ GCE	GCE	SWV ^n^	100 aM–100 pM	31 aM	[43]
MiRNA-21	Ft-ATRP ^o^	Gold electrode	SWV	0.01–100 pM	6.03 fM	[44]
CircRNA ^p^	AuNFs/PNA ^q^	CFME ^r^	CV	10 fM–1 μM	3.29 fM	[45]
MiRNA-141	CMWM ^s^	Gold electrode	DPV	0.5 fM–5 pM	0.26 fM	[46]
MiRNA-21	RNase H endoribonuclease	Gold electrode	DPV	1 pM–100 nM	0.35 pM	[47]
MiRNA-21	MIP/PNA sensor ^t^	Gold electrode	EIS	0.5–5000 pM	0.11 ± 0.04 pM	[48]
MiRNA	Fully FFF 3D-printed sensor ^u^	Screen-printed/gold electrode	SWV	0.001–400 nM	1 pM	[49]

^a^ Dengue virus; ^b^ Triplet nanostructure-mediated dendritic hybridization chain reaction; ^c^ Chronoamperometric; ^d^ Hollow spherical covalent organic frameworks; ^e^ Hepatitis B virus; ^f^ Platinum-decorated cobalt–copper carbon nanocubes; ^g^ Transition metal carbides, nitrides and carbonitrides; ^h^ Glassy carbon electrodes; ^i^ Gold Nanoparticles/Screen-Printed Carbon Electrode; ^j^ Linear sweep voltammetry; ^k^ Palladium nanoparticles; ^l^ Peptide nucleic acid; ^m^ Quadruped DNA walker; ^n^ Square wave voltammetry; ^o^ Ferritin-enhanced atom-transfer radical polymerization; ^p^ Circular RNA; ^q^ Au nanoflower/peptide nucleic acid; ^r^ Carbon-fiber microelectrode; ^s^ Cleat-equipped molecular walking machine; ^t^ Molecularly imprinted polymers/peptide nucleic acids sensors; ^u^ Fully Fused Filament Fabrication 3D-printed electrochemical sensing device.

## Data Availability

All data supporting the findings of this study are available within the article and its Supplementary Materials.

## Supplementary Materials

The following supporting information can be downloaded at https://www.mdpi.com/article/10.3390/s26165308/s1, Figure S1. Schematic construction of LIG electrodes; Figure S2. XRD plot of the AgNFs-SPs probe; Figure S3. The three batches of electrochemical sensors for detecting Norovirus RNA; Table S1. EIS fitting parameters and CV peak currents for each modification and detection step.
