# Supplementary material for: An Ultrasensitive Electrochemical Biosensor for Nucleic Acid Detection Based on Silver Nanoflower-Stem-Loop Probes
Source: Sensors (Basel). 2026 Aug 21;26(16):5308. doi: 10.3390/s26165308 (PMC13517288; doi:10.3390/s26165308)
Supplement: Supplementary file 1 [file sensors-26-05308-s001.zip › sensors-4474105-supplementary.pdf]

Electronic Supplementary Information for

An ultrasensitive electrochemical biosensor for nucleic acid detection based on silver nanoflower-stem-loop probes

Yingying Yuan[1,2,3], Xiaoyu Lei[1], Fengyu Li[1,3], Yuchen Su[1,3], Bo Liu[1,3], Lei Luo[1,*], Hangyu Zhang[1,3,*]

[1] Department of Pediatric Gastroenterology, Women and Children's Hospital of Dalian University of Technology, Dalian, 116000, China

[2] Department of Oncology, Shengjing Hospital of China Medical University, Shenyang, 110004, Liaoning Province, China.

[3] Liaoning Key Lab of Integrated Circuit and Biomedical Electronic System, School of Biomedical Engineering, Faculty of Medicine, Dalian University of Technology, Dalian, 116024, China

Corresponding authors: luolei1818@163.com; hangyuz@dlut.edu.cn.

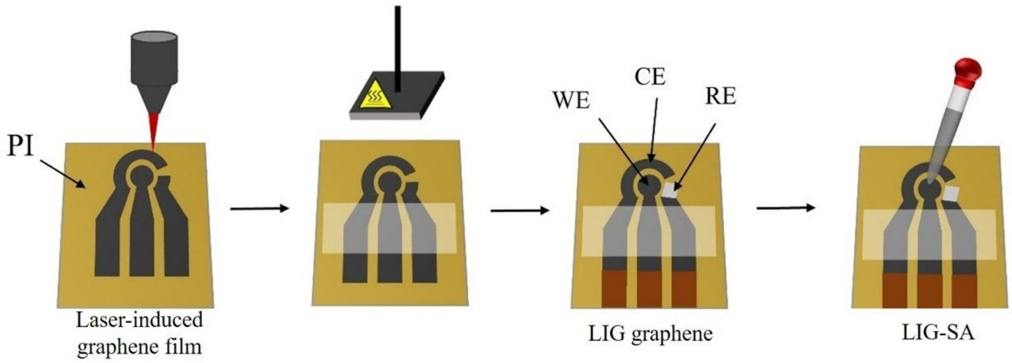

Figure S1. Schematic construction of LIG electrodes.

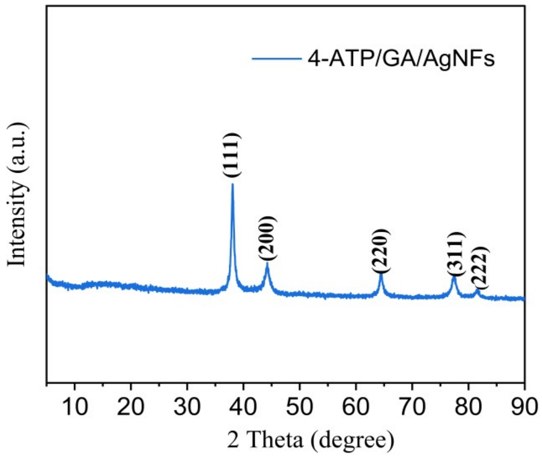

Figure S2. XRD plot of the AgNFs-SPs probe.

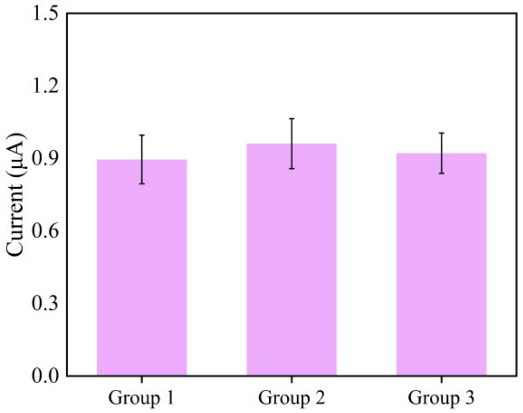

Figure S3. The three batches of electrochemical sensors for detecting Norovirus RNA.

Table S1. EIS fitting parameters and CV peak currents for each modification and detection step.

Electrode modification	R_{ct} (Ω)	CPE-Y_0 ($S \cdot s^n$)	CPE-α	I_{ox} (μA)	I_{red} (μA)
Bare LIG	317	4.46×10^{-3}	0.266	135.80	-92.55
PBASE	394	4.47×10^{-5}	0.755	127.10	-93.08
Streptavidin	458	1.19×10^{-4}	0.821	104.70	-95.74
BSA	470	2.09×10^{-5}	0.839	92.32	-96.77
AgNFs-SP / Target	342	1.38×10^{-4}	0.534	99.24	-86.22
